# Supplementary material for: Single Cell Transcriptomic and Chromatin Profiles Suggest Layer Vb Is the Only Layer With Shared Excitatory Cell Types in the Medial and Lateral Entorhinal Cortex
Source: Front Neural Circuits. 2022 Jan 26;15:806154. doi: 10.3389/fncir.2021.806154 (PMC8826650; doi:10.3389/fncir.2021.806154)
Supplement: Supplementary file 14 [file Data_Sheet_1.ZIP › Exc_vs_Inh_Motifs/homerResults/motif10.similar.html]

motif10

## Information for motif10

G
C
A
T
G
C
T
A
G
C
A
T
C
G
A
T
G
C
T
A
G
C
T
A
G
C
T
A
C
G
A
T
C
G
A
T
G
C
A
T
G
C
T
A
C
G
T
A
  
Reverse Opposite:  

C
G
A
T
C
G
A
T
C
G
T
A
G
C
T
A
G
C
T
A
C
G
A
T
C
G
A
T
C
G
A
T
G
C
T
A
C
G
T
A
C
G
A
T
C
G
T
A
  

|  |  |
| --- | --- |
| p-value: | 1e-241 |
| log p-value: | -5.554e+02 |
| Information Content per bp: | 1.607 |
| Number of Target Sequences with motif | 14298.0 |
| Percentage of Target Sequences with motif | 33.89% |
| Number of Background Sequences with motif | 2888.3 |
| Percentage of Background Sequences with motif | 26.57% |
| Average Position of motif in Targets | 343.7 +/- 241.6bp |
| Average Position of motif in Background | 327.7 +/- 276.9bp |
| Strand Bias (log2 ratio + to - strand density) | 0.0 |
| Multiplicity (# of sites on avg that occur together) | 1.42 |
| Motif File: | file (matrix) reverse opposite |

### Similar de novo motifs found

|  |  |  |  |  |  |  |  |
| --- | --- | --- | --- | --- | --- | --- | --- |
| Rank | Match Score | Redundant Motif | P-value | log P-value | % of Targets | % of Background | Motif file |
| 1 | 0.641 | G T C A C G T A G C A T A G C T G C A T C G T A C G T A C T A G T C A G C G A T C A T G T G A C | 1e-37 | -87.315155 | 0.38% | 0.11% | motif file (matrix) |
